# Global ginseng trade networks: structural characteristics and influencing factors

**DOI:** 10.3389/fphar.2023.1119183

**Published:** 2023-07-10

**Authors:** Yue Fang, Mengxue Tang, Hua Wei, Zhipei Feng, Nianjun Yu

**Affiliations:** ^1^ School of Economics and Management, Anhui University of Chinese Medicine, Hefei, Anhui, China; ^2^ Institute of Chinese Medicine Resources Protection and Utilization, Anhui Academic of Chinese Medicine, Hefei, Anhui, China; ^3^ School of Pharmacy, Anhui University of Chinese Medicine, Hefei, Anhui, China

**Keywords:** ginseng trade, structural characteristics, influencing factors, social network analysis, quadratic assignment procedure

## Abstract

**Background:** Ginseng is a rare and highly valued Chinese materia medica with a rich trading history and has a wide range of application, including medicine, food, healthcare, and daily chemical production. However, the global trade of ginseng exhibits diverse features and uneven development across different countries and regions. Surprisingly, the intricate network relationship and the underlying characteristics and influencing factors of ginseng trade networks remain unexplored.

**Methods:** This study analyzed ginseng trade data obtained from the UN-Comtrade database and used social network analysis to construct global ginseng trade networks. To elucidate the structural characteristics, we analyzed the indicators of the overall network structure and node attributes. Core-periphery analysis is used to examine the evolutionary patterns within the global ginseng trade networks. Furthermore, we apply the quadratic assignment procedure to investigate the impact and relevance of spatial proximity, cultural differences, economic indicators, population size, technological similarity, and institutional distance.

**Results:** The findings reveal that the global ginseng trade networks exhibit typical small-world and scale-free properties, as well as a core-periphery structure. Several core countries, including China, South Korea, Germany, and the United States, exert significant control over both trade volume and trade partners. South Korea and China initially occupied central positions in the export market due to their resource endowments, their prominence has gradually diminished with the ascendancy of Germany and the United States. According to the core-periphery analysis, the ginseng trade has shown a gradual concentration within specific trade groups comprising core and semi-periphery countries, most of which are along the “Belt and Road” religion. We also found that geographic distance and GDP *per capita* exert negative effects on ginseng trade, while factors such as land adjacency, technology and economic gap, population size, and institution similarity play significant positive roles.

**Conclusion:** The global ginseng trade has experienced increasing concentration and close linkage among a limited numbers of participants. It is crucial to pay close attention to the relationship between ginseng industry development and resource conservation. Strategies such as expanding trade channels, implementing trade substitution measures, and optimizing the quality and standards of ginseng products can effectively enhance trade security.

## 1 Introduction

Ginseng (genus*Panax*, family Araliaceae) is a valuable medicinal plant with a rich history of medicinal and edible use. Medical application of ginseng was first found in the Shen Nong Ben Cao Jing (Shen Nong’s Herbal Classic) over 2000 years ago. In 1,596, the Compendium of Materia Medica written by Li Shizen recognized ginseng as a “superior tonic” compared to other herbal remedies (So et al., 2018). The word “ginseng” is originated from a Chinese word meaning “man-herb” while the word “panax” means “cure-all” in Latin, highlighting its reputation as a potent plant capable of treating various diseases (Choi et al., 2015). The root of the ginseng plant is particularly prized for its medicinal properties, containing polysaccharides, saponins, volatile oil, trace elements, organic acid, proteins and other chemical compositions. Ginseng is known for its effectiveness in replenishing primordial Qi, tonifying the spleen and lung, generating body fluids, calming mind and enhancing intelligence (Xin et al., 2021). Modern pharmacology research has revealed that ginseng exerts notable effects on the immune system, central nervous system, cardiovascular system, metabolism, infectious and neoplastic diseases (Chen et al., 2008; Choi et al., 2015; Mancuso and Santangelo, 2017). As a result, ginseng and its products are widely used for eliminating fatigue (Kim et al., 2013), improving cognition (Geng et al., 2010; Scholey et al., 2010), treating depression (Jeong et al., 2015), preventing memory deterioration (Chen and Hui, 2012; Ossoukhova et al., 2015), alleviating diarrhea and shortness of breath (Lee et al., 2012) and combating fatigue (Yennurajalingam et al., 2015). In addition to serve as medical supplies, ginseng is also consumed as dietary supplement and functional food. Its popularity has made ginseng one of the most widely used herbal remedies worldwide.

Thirteen species of ginseng have been identified, but the most commonly utilized are the *Panax ginseng Meyer*, grown in China and Korea, and *Panax quinquefolius*, cultivated in the United States (Virginia, Wisconsin) and Canada (Ontario, Quebec) (Szczuka et al., 2019). The distribution of ginseng is uneven around the world, due to geographical, environmental and economic factors, resulting in significant spatial and temporal difference between major producing nations and others. China is the largest producer of ginseng, followed by South Korea, Canada and the United States and their total production accounts for over 99% of global ginseng production (Baeg and So, 2013). The resource distribution imbalance can be mitigated through close international trade cooperation, which has played a crucial role in compensating for the disparities. Countries with resource advantages enhance their domestic production capacity and earn foreign exchanges, contributing to the sustainable development of their economies. Conversely, countries with limited resources but advanced processing technology and substantial market demand can acquire low-priced raw materials through trade, enabling them to achieve higher profits. Ginseng trade has a long and prosperous history. Historical records show that a considerable portion of ginseng in China originated from ancient Korea during the North and South Dynasties (Zhan et al., 2021). Ginseng exports facilitated economic and trade exchanges between ancient Korea, China and Japan, serving as a vital source of foreign trade income for ancient Korea and a substantial part of China’s tribute trade during Qing dynasty (Flagg, 2021). Ginseng is also regarded as a connection of Chinese-Canadian (Gotlieb, 1985) and Chinese-America relations (Carlson, 1986) dating back to the 18th century. With the growing global interest in alternative medicine and health foods, ginseng trade is projected to reach approximately $17.7 billion by 2030, with a compound annual growth rate (CAGR) of 10.4% from 2022 to 2030 (Fan et al., 2023). The United States ginseng market is estimated to reach $423.9 million by 2030, while the Chinese market is expected to reach $6.3 billion (Baeg and So, 2013; Xiang et al., 2022).

Academic discussion on ginseng trade have primarily focused on the current export structure, export value and export markets (Flagg, 2021; Zhan et al., 2021). However, the global ginseng trade networks (GGTNs) exhibit distinct features and uneven development across different countries or regions. The countries or regions involved in ginseng trade maintain intricate relations, forming a complex system. The trade network characteristics and influencing factors of ginseng trade relationships are still not well understood. Social Network Analysis (SNA) provides a powerful tool for analyzing complex trade relationships by constructing trade networks, extracting core subnets based on trade weights, and studying the structure and attributes between nodes. SNA research has demonstrated typical characteristics such as scale-free distribution, small-world characteristics and high clustering coefficients in international trade relations (Serrano and Boguñá, 2003; Zhong et al., 2014). Trade characteristics have been analyzed by network density, clustering coefficients, and average path length (An et al., 2017). Further investigation carried by Newman and Park (2003); Fagiolo et al. (2009) aimed to study the topological characteristics of trade network, and revealed the core-periphery structure. The dynamics of international trade have also been a key focus for scholars, with previous studies exploring factors affecting trade cooperation using the classical gravity model. Hasson and Tinbergen (1964) were the first to apply the gravity model to international trade research (Leibenstein, 1966). Subsequently, factor endowments (Bergstrand, 1989), economic size (Ristanovic et al., 2020), common borders (Vu et al., 2020) and regional trade agreements (Sui et al., 2021) have been incorporated into the gravity model. The impacts of aging demographics and geographical distance on trade have also been discussed (Liu and Tsai, 2022). However, these studies only partially explain the influencing factors of trade, as they fail to consider the interrelationships between these factors and whether their impacts on trade are influenced by other factors (Fagiolo, 2010; Kabir et al., 2017; Yotov, 2022). Standard statistical procedures are inadequate for parameter estimation and statistical tests, due to the risk of calculating incorrect standard deviations. To address this issue, scholars employ randomized detection methods to test, and quadratic assignment procedure (QAP) is one such approach. QAP compares the similarity of each element in the two matrices, calculates the correlation coefficient between the matrices, and conducts non-parametric test on the coefficient (Dekker et al., 2007; Xu and Cheng, 2016). QAP mitigates problems related to multi-collinearity and structural autocorrelation. Over the past decade, researches combined SNA with QAP have accumulated substantial research experiences and cases, such as crude oil (Guan et al., 2016; Wu and Chen, 2019), fossil energy (Gao et al., 2015; Li et al., 2015), electricity (Ji et al., 2016), etc. However, ginseng trade has not been studied using this approach.

To address this knowledge, this paper aims to use SNA to establish GGTNs and study the network structures and node attributes. Additionally, we adopt QAP to explore the determinants of GGTNs. Our research seeks to answer the following questions: What is the scale of GGTNs? What are their structural characteristics? Whether ginseng networks also have scale-free distribution, small-world characteristics and high clustering coefficients? What are the determinants of GGTNs and how do these factors influence trade relationships? The remainder of this paper is organized as follows: Section 2 introduces the method and data sources. Section 3 focuses on empirical analysis and provides a reasonable explanation of the results. Section 4, 5 concludes discussion and conclusion, respectively.

## 2 Materials and methods

### 2.1 Research methodology

#### 2.1.1 Network construction

In this study, we utilize UCINET 6.504 software to construct a weighted network for GGTNs. Each node in the network represents a country, the edge E_ij_ represents the trade relation between country i and j. If there is no trade relationship between country i and j, then E_ij_ = 0, otherwise, E_ij_ = 1. The trade volume from country i to j is denoted as W_ij_ (Wang et al., 2020). In this study, the trade volume is measured by physical values. Considering the asymmetries in the trade data, we adopt the maximum value as the weight of the edge between countries i and j, following previous studies (Hu et al., 2020).

#### 2.1.2 Statistic indicators

To describe the structural characteristics of GGTNs, this paper sets up two levels of indicators. Firsty, the overall structural features of the network are depicted as follows. The number of network nodes (*N*) represents the number of countries in GGTNs. Density (*D*) describes the level of interconnection between nodes. A higher network density indicates a higher level of commercial activity. Average path length (*L*) is the average number of nodes that need to be traversed to reach from one node to another in the network. The clustering coefficient (*C*) reflects the degree to which a node is connected to its neighboring nodes. If the network exhibits a shorter average path length and a larger clustering coefficient, it indicates the presence of the small-world property (Nobi et al., 2020; Reyes and Laroze, 2022). Secondly, the structural features of the nodes are depicted through their node degree and betweenness centrality. Node degree (*K*) refers to the number of nodes directly attached to a specific node in GGTNs and can be further divided into out-degree and in-degree in a directed network. In this study, the node degree for the year 2010, 2016, and 2021 are ranked from small to large, and the distribution curves reflecting the degree of heterogeneity are plotted (Fu et al., 2021). Betweenness centrality (*BC*) is a measure of the probability that the shortest path between other nodes pass through a particular node in GGTNs, reflecting its control ability. The data are shown in Table 1.

**TABLE 1:** Indicators of the network structure.

Indicators	Equation	Description
Node(*N*)		*N* indicates the total number of network nodes.
Density (*D*)	$D=\frac{M}{N\left(N-1\right)}$	*M*: the number of edges in the network.
Average Path Length (*L*)	$L=\frac{1}{N\left(N-1\right)}\underset{i\neq j}{\sum }d_{ij}$	*d*_*ij*_ reflects the minimum number of edges in all paths from *i* to *j*.
Clustering Coefficient (*C*)	$C=\frac{1}{N}\sum_{i=1}^{N}C_{i}$	*X*_*i*_ is the actual number of connections among *i*’s neighbors
	$C_{i}=2X_{i}/K_{i}\left(K_{i}-1\right)$	
Node-Degree (*K*)	$K_{i}\left(t\right)=K_{i}^{out}+K_{i}^{in}$	*a*_*ij*_ denotes the number from *i* to *j*; *a*_*ji*_ represents the number from *j* to *i*.
	$K_{i}^{in}\left(t\right)=\sum_{j=1}^{N\left(t\right)}a_{ji}\left(t\right)$	
	$K_{i}^{out}\left(t\right)=\sum_{j=1}^{N\left(t\right)}a_{ij}\left(t\right)$	
Betweenness-Centrality(*BC*)	$BC_{i}=\frac{2\underset{jk}{\sum }g_{jk}\left(i\right)/g_{jk}}{n^{2}-3n+2}$	*g*_*jk*_ is the number of shortcuts between *j* and *k*, and *g*_*jk*_(*i*) is the number of shortest paths between *j* and k through *i*.

#### 2.1.3 Core-peripheral analysis

Core-peripheral analysis is used to analyze the evolutionary characteristics of GGTNs, which identifies closely connected centers as well as scattered peripheries. The algorithm for core-peripheral analysis was first proposed by Borgatti and Everett and can be categorized into discrete and continuous model (Borgatti and Everett, 1999; Yanchenko and Sengupta, 2022). In this study, we adopt a continuous core-peripheral model to analyze the evolutionary characteristics of GGTNs by calculating the cores of each node. The specific calculation formula is as follows:

$$\rho =\sum_{ij}a_{ij}\delta_{ij}\left(\delta_{ij}=c_{i}\times c_{j}\right)$$ (1)

In calculation formula, *C*_*i*_ and *C*_*j*_ represent the cores of nodes i and j, respectively. *δ*_*ij*_ represents the element of the pattern matrix *δ* corresponding to the ideal core-edge model, while *a*_*ij*_ represents the element of the actual adjacency weight relation matrix. The correlation index *ρ* measures the correlation between the pattern matrix and the actual adjacency matrix. When *ρ* reaches the maximum value, *δ* represents the edge-core structure matrix that closely approximates the actual situation and corresponds to the nearest quasi-ideal model (Elliott et al., 2020).

#### 2.1.4 QAP analysis and factor selection

QAP analysis is a randomized detection method that consists of correlation analysis and regression analysis. The correlation analysis examines the relationship between each influencing factor and the trade network, while the regression analysis investigates the statistical significance and magnitude of these influencing factors (Liu, 2004). The QAP algorithm proceeds in three steps. Firstly, it calculates the Pearson correlation coefficient between corresponding cells of the two data matrices. Secondly, it randomly permutes rows and columns of one matrix and recalculates the correlation and other measures. Lastly, step 2 is repeated thousands of times to determine the proportion of times the randomly generated measure is equal to or greater than the observed measure calculated in step 1.

The first factor influencing GGTNs is spatial proximity, which includes geographic distance and common border. The geographic proximity between economic entities plays a significant role in determining trade linkages, as it helps to reduce transaction costs. Studies conducted by Anderson and Wincoop have demonstrated an inverse relationship between trade volume and geographical distance (Anderson and Van Wincoop, 2003). Additionally, a common land border is considered as a crucial factor in measuring trade cost, serving as a proxy variable for geographical distance. The second factor is cultural similarities. Language and religion, as core components of cultural connotations, directly impact the way and cost of communication in international trade. Shared cultural contexts facilitate a reduction in information acquisition costs and cognitive blind spots on both sides, leading to improved credit enhancement and increased international trade (Walker, 2018). The third factor is the economy and population. Countries with similar economies and populations often share similar consumer preferences. The level of personal income also influences consumption pattern. Duan et al. (2021) hold the view that food consumption patterns change with rising incomes, leading to an increased *per capita* food consumption. The fourth factor is technological similarity. Countries with high technological similarity are more likely to establish cooperative relations and there is active trade between countries engaged in close technical cooperation. The final factor is institutional distance, which can result in trade friction. The diversity of the national drug surveillance system means that ginseng trade has gone beyond commercialization and is also influenced by political and cultural dynamics. The International Regulatory Cooperation for Herbal Medicines (IRCH), established in 2006, is a global organization of medical plant regulators. In this study, IRCH membership is used as a proxy variable for institutional distance. The definitions and data sources of these factors are described in Table 2.

**TABLE 2:** Variables, description, and data source of QAP model.

Variable	Description	Source
Geographical Distance	Absolute value of the difference in distance between the two capitals	http://www.cepii.fr
Land adjacency	Whether there is a common geographical boundary contiguity	http://www.cepii.fr
GDP	Absolute value of GDP difference	World Bank Open Data | Data
GDP_per	Absolute value of GDP_per difference	World Bank Open Data | Data
Technology	Absolute value of the difference in ginseng patent between two countries	https://www.incopat.com
IRCH members	Whether to join the IRCH organization as an independent state	https://irch.org/
Religion	Whether there is a common religious.	CEPII-Accueil
Language	Whether there is a common language	CEPII-Accueil
Population	Absolute value of population difference	World Bank Open Data | Data

Based on the above analysis, we propose the following hypotheses on the factors influencing GGTNs:

Hypothesis 1 (H1). Countries that are geographically closer or with common geographical boundaries are more likely to trade with each other.

Hypothesis 2 (H2). Countries shared common culture background, such as language or religion, are more likely to trade with each other.

Hypothesis 3 (H3). Countries with similar economies and populations are more likely to trade with each other.

Hypothesis 4 (H4). Countries with high technological similarity are more likely to establish cooperative relations.

Hypothesis 5 (H5). IRCH members are more likely to trade with each other.

According to the above analysis, the model constructed in this study is as follows:

T = f (Diff_distance, Binary_border, Binary_language, Binary_religion, Diff_GDP, Diff_GDP_per, Diff_population, Diff_ technology, Binary_IRCH)

where the dependent variable *T* represents the matrix of GGTNs, *Diff_distance, Diff_GDP, Diff_GDP_per, Diff_population and Diff_technology* are matrices that represent the absolute differences in the corresponding indexes. These five variables are standardized by the columns of the matrix. *Binary_border, Binary_language, Binary_religion, Binary_IRCH* are binary matrices. If two countries are the same, the value takes 1, otherwise it takes 0.

### 2.2 Data resource

To investigate the dynamic changes in ginseng trade, trade data from the UN-Comtrade database spanning from 2010 to 2021 were extracted. Data prior to 2010 were not included in the analysis due to the limited number of countries involved in ginseng trade and the small trade volume during that period. The specific trade data for ginseng root is identified by the HS code HS121100. Trade related to ginseng extracts was not analyzed as there was no corresponding HS code available. In order to present the main structure of GGTNs more clearly, certain countries with low trade volumes were excluded from the analysis. Re-export and re-import quantities were not considered, because of their tiny proportion in the overall trade. China and Hong Kong China were separately included in this study. Table 2 presents the variables used in QAP analysis. Data on GDP, GDP_*per capita* and total population were acquired from the World Bank database. Geographical distance, land boundary, language and religion were obtained from the Cep II database. Technology differences were measured by the absolute value of the difference in ginseng patents. As ginseng is a botanical medicine, the inclusion of independent countries in IRCH organization is also considered in the model.

## 3 Results

### 3.1 A synopsis of GGTNs

**FIGURE 1:**
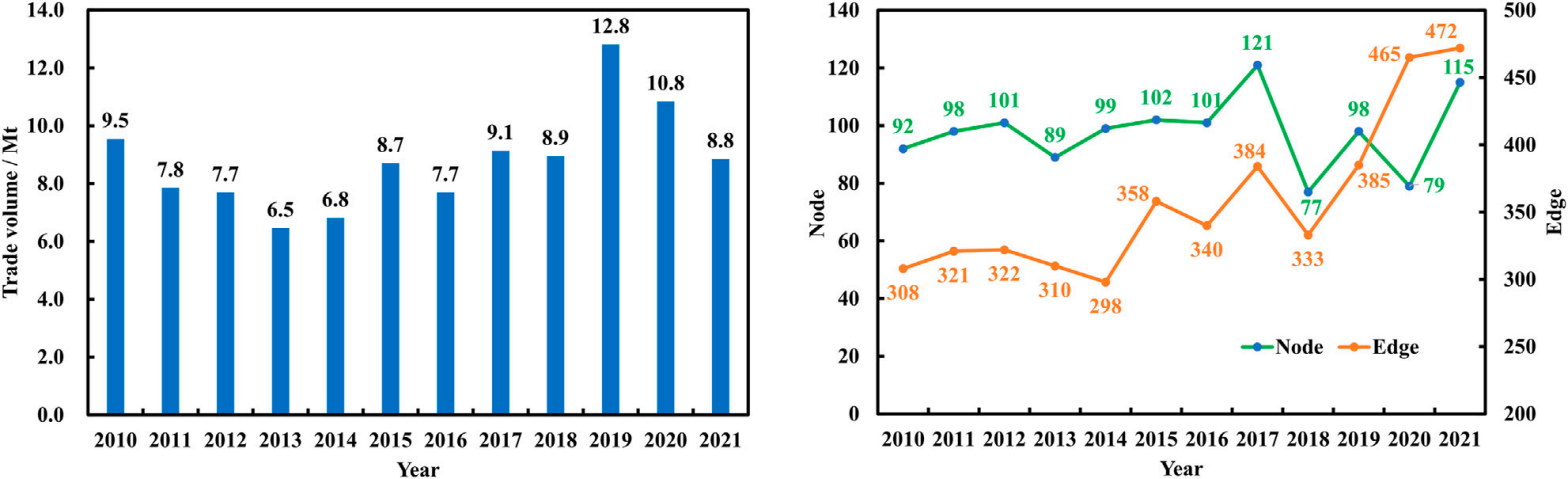
Changes in trade volume (million tonnes), numbers of nodes and edges from 2010 to 2021.

**FIGURE 2:**
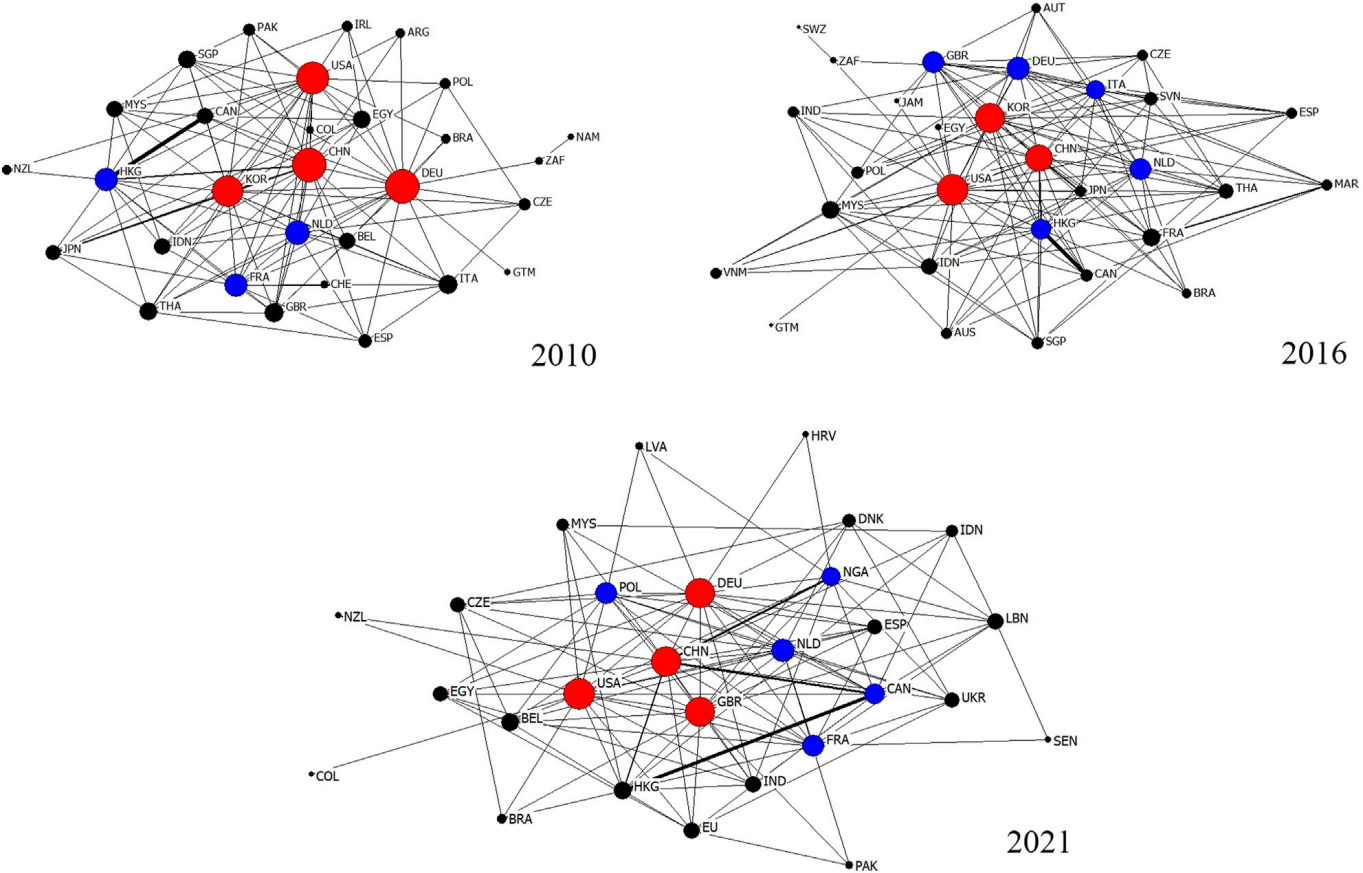
GGTNs in 2010, 2016, and 2021. Note: Each node denotes a country using ISO alpha-3 abbreviation. See Supplementary Appendix Table SA4. for the country codes.

Over the past decade, the trade volume of ginseng has remained relatively stable. This can be attributed to the stringent growing conditions of ginseng and the limited number of producers. The trade volume initially increased from 9.5 million tons in 2010 to a peak of 12.8 million tons in 2019, but decreased to 8.8 million tons in 2021. Figure 1 illustrates the scale of GGTNs. The number of countries participating in GGTNs has steadily increased from 92 in 2010 to 115 in 2021, reaching a peak of 121 in 2017. Moreover, the number of edges connecting these nodes has also grown from 308 in 2010 to a maximum of 472 in 2021. This indicates that the participating countries have become more closely interconnected over the past decade. To provide a clearer representation of the GGTNs structure, some countries with low trade volume were excluded from the analysis. In this study, the top 30 countries with trade volume in 2010, 2016, and 2021were selected to construct GGTNs. Figure 2 graphically represents the global structure of the international ginseng trade network. Each node in the figure represents a country, and the edges denote trade relations between two countries.

**FIGURE 3:**
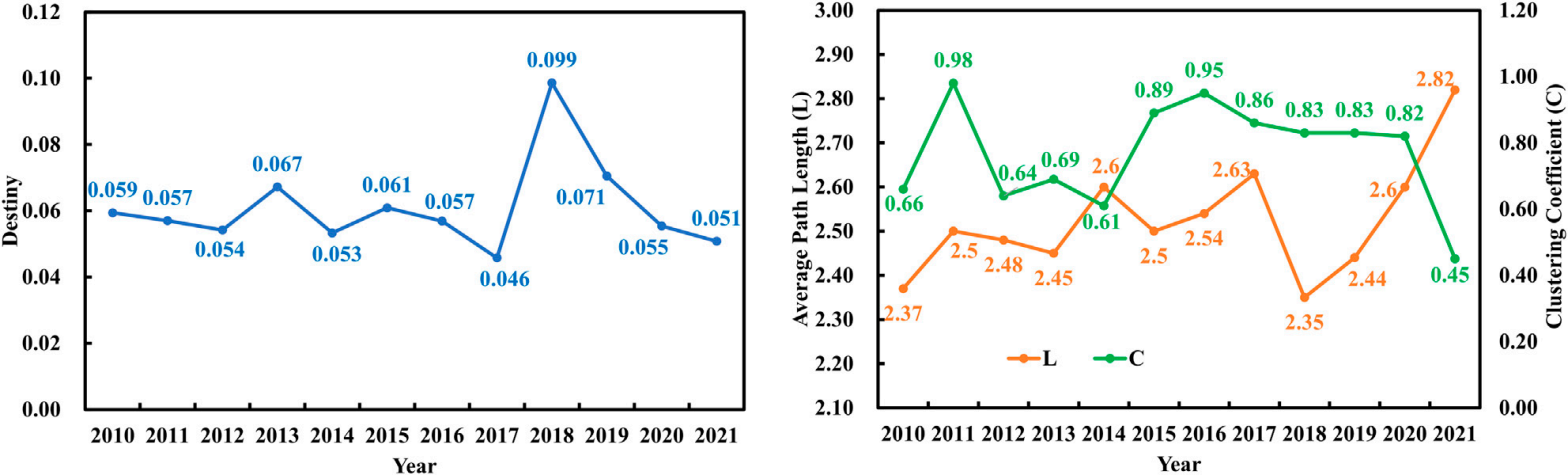
Network density (*D*), average path length (*L*), and clustering coefficient (*C*) of GGTNs from 2010 to 2021.

Figure 3 demonstrates the changes in network density, average path length (*APL*), and clustering coefficient (*C*) of GGTNs over time. The density exhibited a general growth trend with fluctuations. The highest network density observed was 0.099 in 2018, while the lowest density was record as 0.046 in 2017. This result indicates a relatively stable ginseng trade. The *APL* value ranged from 2.35 to 2.82, which implied that any two nodes within the GGTNs could be connected through an average of less than three nodes. Simultaneously, the *C* value ranged from 0.45 to 0.98 and exhibited a continuous increase from 2013 to 2021. Notably, the maximum *APL* value was more than six times greater than the minimum *C* value. The opposite trends of them indicate that GGTNs has typical small-world characteristics.

### 3.2 Nodes attribution

In this study, the node degree was ranked from small to large, and the top 10 nodes in 2010, 2016 and 2021were identified and presented in Table 3. Although the ranking of node degrees varied over times, certain key countries such as China, South Korea, Germany, the United States and Britain consistently ranked among the top positions. The United States and Germany maintained their strong positions in import market, indicating their significant market demand for ginseng. South Korea and China ranked first and second, respectively, in the out-degree rankings in 2010 and 2016, highlighting their substantial export advantage due to their notable resource endowments. However, Germany emerged rapidly and surpassed South Korea to become the top-ranked country in 2021. The results of betweenness centrality revealed that China and South Korea were the main connector hubs of GGTNs in 2010 and 2016. However, in 2021, Lebanon and the United States replaced them in this role. The above analysis focused on the number of trading partners for each node. Further research on the top ten trade routes based on trade volume indicated that countries with a higher numbers of trading partners also tended to have higher transaction volumes. The data can be founded in Table 4.

**TABLE 3:** The top 10 nodes of GGTNs in 2010, 2016 and 2021.

2010			2016			2021		
In-degree	Out-degree	Betweenness	In-degree	Out-degree	Betweenness	In-degree	Out-degree	Betweenness
United States	Korea	China	Germany	Korea	Korea	Germany	Germany	Lebanon
Germany	China	Korea	United States	China	China	United States	Lebanon	United States
Netherlands	Germany	United States	United Kingdom	Germany	United States	United Kingdom	EU	EU
United Kingdom	United States	United Kingdom	Netherlands	United Kingdom	Germany	China	Netherlands	Germany
Japan	United Kingdom	Canada	France	Netherlands	United Kingdom	Poland	China	United Kingdom
China	Italy	France	Singapore	United States	South Africa	France	United States	China
India	France	Germany	China	France	Netherlands	Netherlands	Poland	Netherlands
France	Hong Kong	India	Hong Kong	Slovenia	France	Canada	United Kingdom	El Salvador
Spain	Indonesia	South Africa	Spain	Hong Kong	Thailand	Lebanon	France	France
Belgium	Pakistan	India	Slovakia	Thailand	Spain	EU	Canada	India

**TABLE 4:** The top 10 trade routes of ginseng in 2010, 2016 and 2021.

2010	2016	2021
Routes(volume, kilotons)	Routes (volume, kilotons)	Routes (volume, kilotons)
Canada→Hong Kong(2,895.2)	Canada→Hong Kong(2,464.2)	Canada→Hong Kong (1,472.5)
China→Japan (917.4)	Morocco→Belgium(706.4)	Canada→China (1,378.3)
China→Hong Kong(668.2)	Pakistan→Egypt (630.9)	Nigeria→China (1,370.6)
Pakistan→Egypt(533.9)	China→Hong Kong(561.3)	Hong Kong→China (388.7)
Pakistan→India(436.5)	Hong Kong→China(481.9)	Nigeria→Italy (338)
China→Italy (278.1)	China→Japan (416.9)	Nigeria→Greece (323)
Hong Kong→China(241.6)	Morocco→France (325.8)	China→New Zealand (256.5)
USA→China (197.8)	USA→China (175.8)	USA→Hong Kong (256.4)
China→Germany(189.3)	Indonesia→Vietnam (132.7)	Ukraine→Netherlands (192.9)
Hong Kong→CAN(158.2)	China→Germany (129.3)	China→Malaysia (188.5)

**FIGURE 4:**
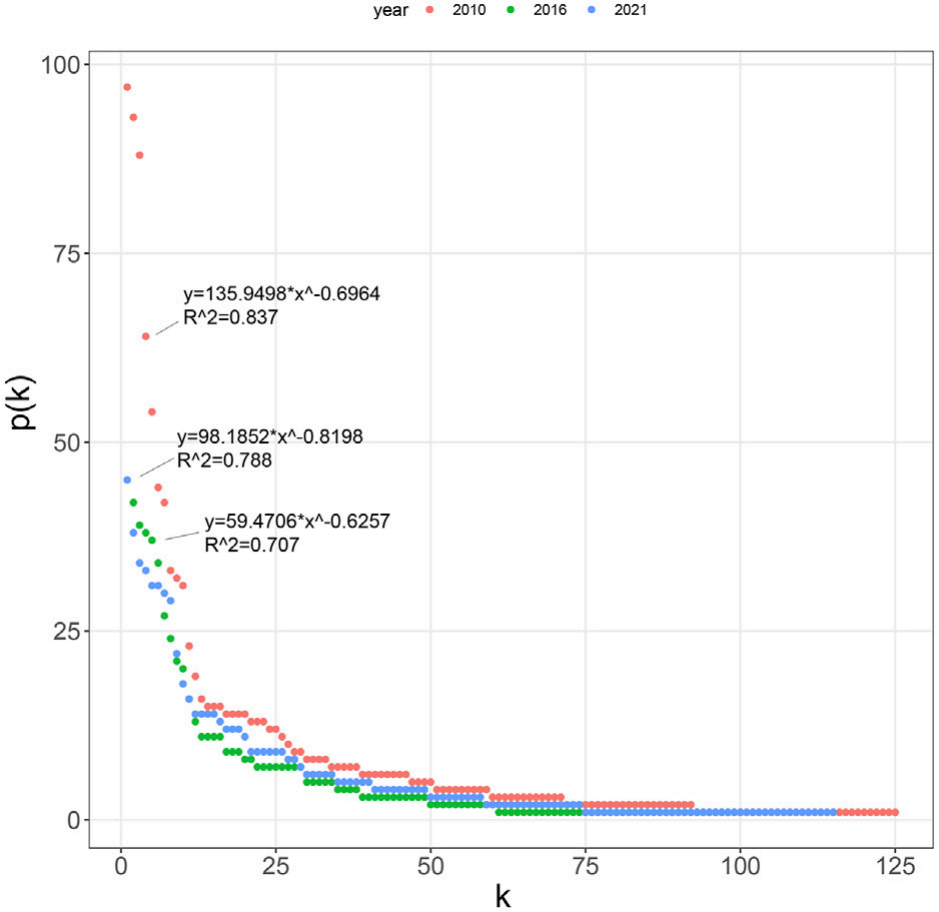
Dstribution curve of node degree in 2010, 2016, and 2021.

Power function fittings were conducted to analyze the node degree distribution in GGTNs and both fittings passed the significance test. A comparison of the node degree distribution curves for 2010, 2016, and 2021 revealed a typical “long-tail” distribution pattern. This indicates that a few nodes in the networks had high degree values, while the majority had small and similar degree values (Figure 4). The node degree distribution followed a power-law distribution indicating the ginseng trade exhibits scale-free characteristics. However, the feature was weakening over time, as evidenced by the decreased in the power ratio fitting value (R^2^) since 2010. This suggests that GGTNs may become more decentralized and diversified in the future.

### 3.3 Core-periphery structure analysis

The participating countries in GGTNs for the years 2010, 2016, and 2021 were categorized into three groups based on their core values. Countries with core values greater than 0.2 were classified as core countries, those between 0.1 and 0.2 were classified as semi-periphery countries, and those below 0.1 were classified as periphery countries. The analysis revealed a significant decline in the number of core countries, with six core countries in 2021, compared to nine in 2016 and seven in 2010. As shown in Table 5, Germany, the United States, and China firmly hold core positions, with their core value greater than 0.3. Germany, in particular, has become the top-ranking country in terms of both core value and node degree. Conversely, countries like South Korea and Hong Kong China have changed from being core countries to semi-periphery countries, indicating a decline in their positions. On the other hand, there has been a substantial increase in the number of semi-periphery countries, with eleven in 2010, fourteen in 2016 and seventeen in 2021. This trend is noteworthy due to the large numbers and the potential market opportunities these countries represent. Regional variations were observed within the semi-periphery countries, with a decrease in core values for Asian countries such as Japan, Singapore, Thailand, and an increase for European countries like Spain and Italy. Most of the core and semi-periphery countries, such as China, Japan, Singapore, Thailand are along the “Belt and Road” (BRI). It is worth investigating the factors influencing GGTNs, as the geographic proximity and cultural similarities, particularly among countries along the BRI initiative, may play a significant role.

**TABLE 5:** The top 10 countries in terms of core value.

Rank	2010		2016		2021	
	Country/Region	Core value	Country/Region	Core value	Country/Region	Core value
1	China	0.359	Korea	0.360	Germany	0.332
2	Korea	0.338	United States	0.331	United States	0.299
3	United States	0.314	China	0.323	China	0.289
4	Germany	0.313	Netherlands	0.252	Netherlands	0.273
5	Netherlands	0.268	Germany	0.245	United Kingdom	0.267
6	France	0.245	United Kingdom	0.241	Poland	0.249
7	United Kingdom	0.221	Hong Kong	0.231	France	0.197
8	Thailand	0.195	Italy	0.221	EU	0.179
9	Italy	0.180	France	0.215	Canada	0.164
10	Singapore	0.177	Indonesia	0.195	Spain	0.161

### 3.4 Analysis of influencing factors

#### 3.4.1 QAP correlation analysis

In this study, a correlation analysis was conducted between the ginseng trade matrix and its impact factors for the years 2010, 2016, and 2021. A total of 5,000 random permutations were performed for statistical analysis.

Table 6 presents the results of the correlation analysis. It is evident that geographic distance, land border, GDP, technology differences and membership of IRCH organization had significant effects on GGTNs at a 1% significance level. Among these factors, geographic distance showed a negative correlation with the network, suggesting that greater distance between countries, the lower trade volume. Additionally, GDP_per also exhibited a negative correlation with the network. The correlation coefficients for population, religion and language were relatively small, and their significance varied sporadically. This implies that these factors had limited overall influence on the ginseng trade network in the mentioned years.

**TABLE 6:** QAP correlation analysis results.

Variables	2010	2016	2021
Diff_Geographic distance	−0.218^c^	−0.254^c^	−0.283^c^
Diff_Land borders	0.293^c^	0.201^c^	0.232^c^
Diff_GDP	0.164^b^	0.208^a^	0.258^c^
Diff_ GDP Per	−0.022	−0.074^a^	−0.050
Diff_Population	0.132^a^	0.087	0.166^b^
Diff_Religion	0.128^b^	0.001	0.041
Diff_Language	0.134^b^	0.032	0.082^b^
Diff_Technology	0.402^c^	0.312^c^	0.285^c^
Diff_IRCH organization	0.200^b^	0.219^c^	0.215^c^

^a^ *p* < 0.1.

^b^ *p* < 0.05 and.

^c^ *p* < 0.01.

#### 3.4.2 QAP regression analysis

To further explore the statistical significance of the explanatory variables, QAP regression analysis was conducted. The regression results for the year 2010, 2016 and 2021 are shown in Table 7.

**TABLE 7:** Results of QAP regression.

Variables	2010	2016	2021
Diff_Geographic distance	−0.158^c^	−0.264^c^	−0.352^c^
Diff_Land borders	0.179^c^	0.080^b^	0.063^a^
Diff_GDP	−0.038	0.052	0.083^a^
Diff_ GDP Per	0.054	0.037	−0.088^b^
Diff_Population	0.019	−0.019	0.126^c^
Diff_Religion	0.100^b^	−0.035	−0.024
Diff_Language	0.060	0.052^a^	0.026
Diff_Technology	0.424^c^	0.284^c^	0.256^c^
Diff_IRCH organization	0.061	0.108^b^	0.133^c^
R^2^	0.283	0.202	0.258
Adj-R^2^	0.270	0.193	0.251

^a^ *p* < 0.1.

^b^ *p* < 0.05 and.

^c^ *p* < 0.01.

The analysis revealed that geographic distance and land adjacency were consistently significant factors with the greatest impact on GGTNs over the long term. Specifically, the coefficient of geographic distance was statistically negative at a 1% critical level for all 3 years, suggesting that the shorter geographic distance, the higher level of trading volumes. This finding aligns with the previous literature that high transportation costs encourage countries to trade with partners located closer to them. The significant impact of land borders suggests the presence of a noticeable “boundary effect” in ginseng trade. Countries in close proximity may share similar cultures and lifestyles, leading to lower construction and management costs and more flexible railway transportation. For example, the analysis of the top 10 trade routes revealed that China has maintained increasingly close trade relation with its neighbors. However, the influence of land borders may be diminishing due to the availability of alternative transportation modes such as marine and air freight.

Furthermore, in the year 2021, the coefficient of economic proximity and population showed a positive and statistically significant relationship with GGTNs, whereas in 2010 and 2016, these variables did not demonstrate significant effects. This suggests that countries with higher levels of economic development and significant population disparities tend to engage in more frequent ginseng trade. The underlying reason for this finding could be attributed to the resource-intensive and labor-intensive nature of ginseng industry. Countries with different economic scale and population size possess diverse comparative advantages, which can be mutually beneficial through international trade. The availability of complementary resources and labor forces encourages trade interactions between countries with different economic and population characteristics, leading to increased ginseng trade volumes.

The influence of economic proximity and population on GGTNs is consistent with the characteristics of the top 10 trade nodes, which predominantly include developed countries such as China and the United States. However, an interesting finding in 2021 was opposite impact of GDP_per compared to GDP in 2021. Countries with similar GDP_*per capita* are more likely to establish trade relationships for ginseng products. One possible explanation for this is that people with similar income level tend to have similar consumption behaviors and habits. Hence, countries with comparable level of GDP_*per capita* may share common preferences and demand patterns, facilitating trade collaborations in the ginseng industry.

Religion and language were found to be positive factors influencing GGTNs, but their significance was observed only in 2010, and showed a declining trend over time. This finding implies that ginseng trade initially benefited from similar culture backgrounds, but with the advancement of economic globalization, cultural barriers in GGTNs have been progressively diminished. One the other hand, technology similarity exhibited a positive and long-term significant influence at a 1% level. This result is consistent with previous studies indicating that technological proximity facilitated trade and scientific research (Milani, 2020). As ginseng is a medicinal plant, its medicinal value can be better understood and appreciated by members of the IRCH organization, thus facilitating its promotion, application and supervision. The factor analysis showed that membership in the IRCH organization had a significant positive effect on ginseng trade, and its effect has increased over time.

#### 3.4.3 Robustness test

To ensure the robustness of the QAP regression results, a variable exclusion test was conducted to assess the stability of the findings. The results of the variable exclusion tests in 2010, 2016 and 2021 demonstrated the robustness of the QAP regression analysis. When one variable was removed, the regression coefficient and significance levels of the remaining variables generally remained consistent with the original results.

There are several minor changes observed in the variable exclusion tests, in 2010, when the technology variable was excluded, the regression coefficient of GDP difference changed from negative to positive and became significant. In 2016, when technology variable was excluded, the coefficient of population changed from negative to positive, but it was not statistically significant. In 2021, when geographic distance variable was excluded, the coefficient of religion difference varied from negative to positive, but it was not statistically significant. The detailed results of the robustness test are presented in Supplementary Appendix Table SA1–SA3, due to space limitations in the main article.

## 4 Conclusion and discussion

### 4.1 Conclusion

In this study, we use ginseng trade data spanning 2010 to 2021 to construct global ginseng trade networks and analyze its structural characteristics using social network analysis. We further investigate the influencing factors of ginseng trade by quadratic assignment procedure. There are several interesting findings.

Firstly, SNA research has demonstrated typical characteristics such as scale-free distribution, small-world characteristics and high clustering coefficients in ginseng trade relations. This finding is consistent with the characteristics observed in international trade networks (Serrano and Boguñá, 2003; Zhong et al., 2014). They indicate that ginseng trade has been concentrated among certain active participating countries over the past decade. Additionally, core-periphery analysis was conducted to further investigate the specific trade groups within the ginseng trade network. This analysis confirmed the presence of distinct trade groups consisting of core and semi-periphery countries.

Secondly, several core countries, including China, South Korea, Germany, and the United States, play a dominant role in terms of both trade volume and trade partners within the ginseng trade networks. Initially, South Korea and China held central positions in the export market due to their resource endowments. However, their prominence has gradually diminished with the rise of Germany and the United States. Furthermore, core-periphery analysis found that most of the active participating countries in the ginseng trade are along the “Belt and Road” religion.

Finally, QAP results reveal that geographic distance and GDP *per capita* have negative impacts on ginseng trade, indicating that countries that are geographically distant and have lower GDP *per capita* tend to engage in less trade in ginseng. On the other hand, factors such as land adjacency, technology and economic gap, population size, and institution similarity were found to have significant positive effects on ginseng trade. These result helps us to better understand the complex ginseng trade relationships and thus to formulate trade policies.

### 4.2 Theoretical contributions and limitations

In this paper, we provide a novel perspective to study the ginseng trade relations. Importantly, our study reveals the typical characteristics including scale-free distribution, small-world characteristics and high clustering coefficients in ginseng trade relations. This finding extends the literature on trade in ethnic medicine. Also, our findings suggest that GGTNs have become increasingly concentrated and interconnected among a few exporters with abundant resources, as well as consumers who value intellectual property rights and brand advantages. Interesting, the resource endowments of these exporters have been gradually eroded due to advancements in deep processing technology and most of the active participating countries are along the “Belt and Road” religion. Additionally, our study enriches the theoretical literature on the dynamics of international trade by confirming the impact of geographic distance, GDP *per capita*, land adjacency, technology and economic gap, population size, and institution similarity.

Despite the contributions herein, this work has several limitations. The study on GGTNs should not only focus on the national level, but also extend to regional alliances. Other factors such as medical insurance policy differences that have an impact on the ginseng trade are yet to be explored.

### 4.3 Practical implications

#### 4.3.1 Commerce and conservation

It is crucial to highlight the relationship between the responsible use of resources and sustainable trade in ginseng trade. The rational utilization of resources plays a pivotal role in ensuring the long-term sustainability of the economy, and the level of economic development significantly influences the pattern of resource exploitation. It is imperative that trade activities do not jeopardize the survival of species or contribute to their extinction.

We firmly advocate for the pursuit of sustainable development in ginseng trade, similar to the approaches adopted in food and energy trade. This necessitates the establishment of a consensus and cooperation among all participating countries engaged in ginseng trade. Furthermore, it is essential for international organizations such as the IRCH organization to actively engage and implement oversight measures to ensure sustainable practices are followed.

#### 4.3.2 More diversified products, more secure trade

Expanding trade channels and adopting trade substitution measures are essential to improve trade security, given the similar market structure and high barriers in ginseng trade. Currently, the export of high value-added and high-tech ginseng patent medicines, healthcare products and cosmetics is limited. Certain ginseng medicinal products, such as ginseng herbal tablets and single-herb granules face legal restrictions in some countries because of legal barrel (Moorhouse et al., 2021). To bridge the gap between the Chinese and Western cultures as well as their dissimilar medical systems, the government should leverage business cooperation and culture exchanges to explore the overseas markets, especially potential semi-periphery markets. It is crucial to foster an understanding of traditional Chinese medicine culture as a precondition for the acceptance of Chinese medicine (Yu et al., 2022). Furthermore, it is essential for governments and enterprises to recognize the importance of expediting legislation, strengthening high-level contacts and dialogs, and studying and using existing laws, regulations, and traditional drug management practices. These actions are necessary to expand offshore markets in a comprehensive manner.

#### 4.3.3 Standardized production and quality improvement

To enhance the quality and safety of ginseng products and improve market competitiveness, the implementation of standardized production and brand management practices is crucial. Currently, more than 60% of Chinese medicines face barriers to entry in foreign markets due to “green barriers” (Cunningham et al., 2018). To address concerns and doubts regarding the safety and efficacy of ginseng, it is essential to establish a quality standard system that aligns with international standards across various stages, including the sourcing of herbs, extraction and separation processes, research, development, production, and quality control.

Furthermore, fostering exchanges and cooperation between Traditional Chinese Medicine colleges, research institutions, clinical institutions, and peers should be encouraged and supported. As the efficacy of TCM becomes widely recognized, an increasing number of research institutions are expected to contribute to deeper investigations on further processed products and high-value ginseng products.

## Data Availability

The original contributions presented in the study are included in the article/Supplementary Material, further inquiries can be directed to the corresponding author.

## Abbreviations

SNA, Social Network Analysis; QAP, Quadratic Assignment Procedure; GGTNs, Global Ginseng Trade Networks; CAGR, Compound annual growth rate; N, Node; D, Density; L, Average Path Length; C, Clustering Coefficient; K, Node-Degree; BC, Betweenness Centrality.
