# Supplementary figures and images for: Global ginseng trade networks: structural characteristics and influencing factors
Source: Front Pharmacol. 2023 Jul 10;14:1119183. doi: 10.3389/fphar.2023.1119183 (PMC10364324; doi:10.3389/fphar.2023.1119183)

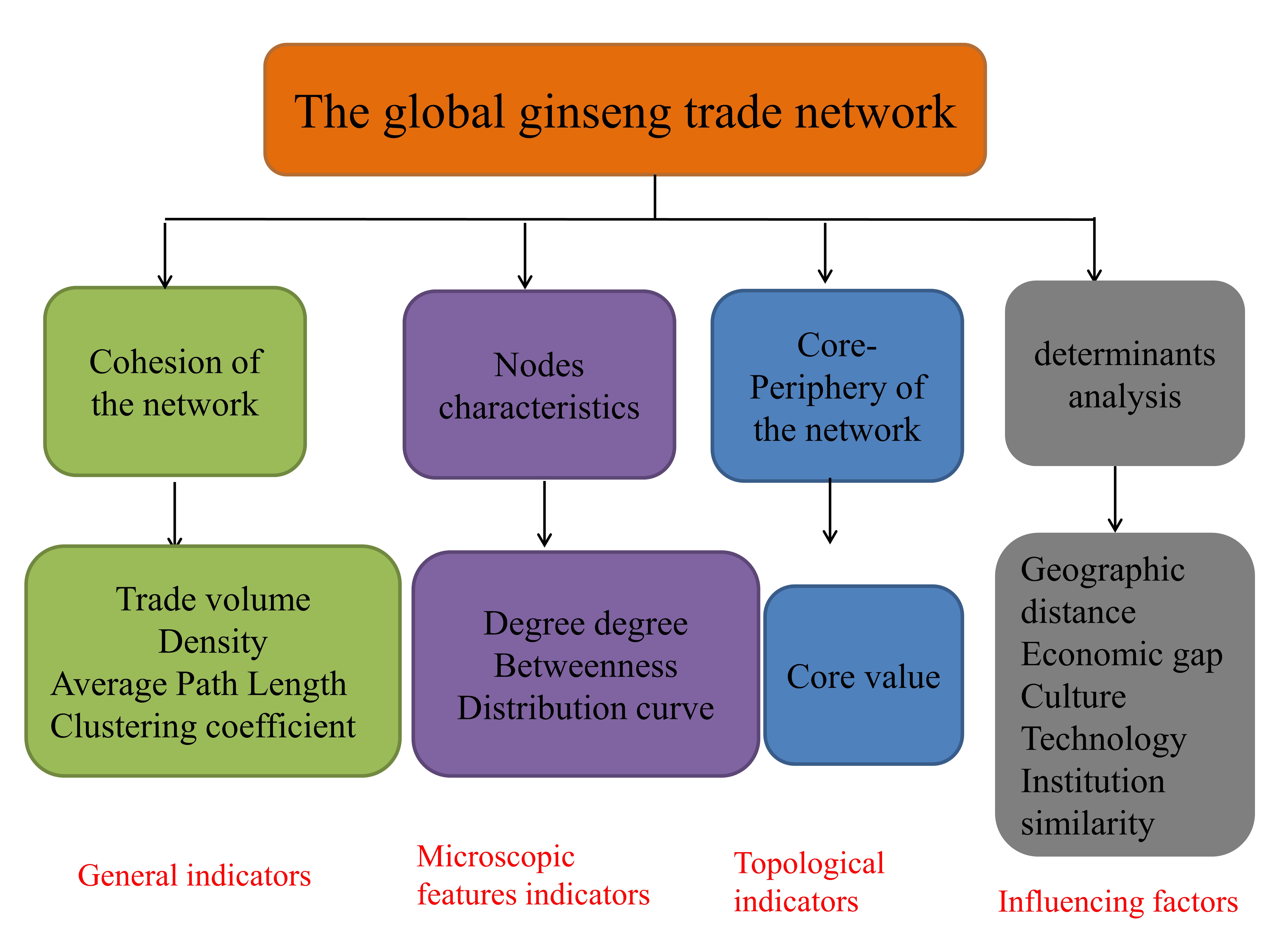

Supplement: Supplementary file 1 [file Image1.JPEG]
