# Supplementary material for: Global ginseng trade networks: structural characteristics and influencing factors
Source: Front Pharmacol. 2023 Jul 10;14:1119183. doi: 10.3389/fphar.2023.1119183 (PMC10364324; doi:10.3389/fphar.2023.1119183)
Supplement: Supplementary file 2 [file DataSheet1.docx]

**Supplementary material**

**Global Ginseng Trade Networks: Structural Characteristics and Influencing Factors**

**Yue Fang^1^, Mengxue Tang^1^, Hua Wei^2^, Zhipei Feng^1^, Nianjun Yu^3*^**

**Acknowledgements**

This study was supported by Major Science and Technology Special Project of Anhui Province, Grant/Award Number: 202003a07020011; Philosophy and Social Science Planning Project of Anhui Province , Grant/Award Number:2022AH050424.

**Abbreviations**

SNA Social Network Analysis

QAP Quadratic Assignment Procedure

GGTNs Global Ginseng Trade Networks

IRCH International Regulatory Cooperation for Herbal Medicines

BRI The Belt and Road

CAGR Compound annual growth rate

*N* Node

*D* Density

*APL* Average Path Length

*C* Clustering Coefficient

*K* Node-Degree

*BC* Betweenness Centrality

**Table A.1.** Results of the variable exclusion test in 2010.

| Variables | Model 1 | Model 2 | Model 3 | Model 4 | Model 5 | Model 6 | Model 7 | Model 8 | Model 9 |
| --- | --- | --- | --- | --- | --- | --- | --- | --- | --- |
| Diff_Geographic distance | - | -0.241^***^ | -0.159^***^ | -0.159^***^ | -0.156^***^ | -0.175^***^ | -0.165^***^ | -0.136^**^ | -0.150^***^ |
| Diff_Land borders | 0.251^***^ | - | 0.181^***^ | 0.169^***^ | 0.181^***^ | 0.176^***^ | 0.185^***^ | 0.218^***^ | 0.184^***^ |
| Diff_GDP | -0.047 | -0.048 | - | -0.429 | -0.035 | -0.053 | -0.039 | 0.170^**^ | -0.032 |
| Diff_ GDP Per | 0.058 | 0.026 | 0.056 | - | 0.055 | 0.036 | 0.052 | 0.058 | 0.058 |
| Diff_Population | 0.008 | 0.030 | 0.014 | 0.021 | - | 0.015 | 0.011 | 0.093 | 0.024 |
| Diff_Religion | 0.124^**^ | 0.095^**^ | 0.105^**^ | 0.090^**^ | 0.100^**^ | - | 0.105^**^ | 0.095^*^ | 0.115^**^ |
| Diff_Language | 0.075^*^ | 0.073^*^ | 0.061 | 0.059 | 0.058 | 0.067^*^ | - | 0.072 | 0.068^*^ |
| Diff_Technology | 0.416^***^ | 0.443^***^ | 0.406^***^ | 0.425^***^ | 0.429^***^ | 0.423^***^ | 0.427^***^ | - | 0.430^***^ |
| Diff_IRCH organization | 0.045 | 0.071 | 0.058 | 0.064 | 0.063 | 0.084^*^ | 0.069 | 0.088 | - |
| R^2^ | 0.265 | 0.260 | 0.282 | 0.280 | 0.283 | 0.274 | 0.280 | 0.168 | 0.280 |
| Adj-R^2^ | 0.254 | 0.248 | 0.271 | 0.269 | 0.272 | 0.263 | 0.268 | 0.155 | 0.269 |

^*^ p < 0.1, ^**^ p < 0.05 and ^***^ p < 0.01.

**Table A.2.** Results of the variable exclusion test in 2016.

| Variables | Model 1 | Model 2 | Model 3 | Model 4 | Model 5 | Model 6 | Model 7 | Model 8 | Model 9 |
| --- | --- | --- | --- | --- | --- | --- | --- | --- | --- |
| Diff_Geographic distance | - | -0.293^***^ | -0.264^***^ | -0.266^***^ | -0.265^***^ | -0.260^***^ | -0.264^***^ | -0.243^***^ | -0.258^***^ |
| Diff_Land borders | 0.175^***^ | - | 0.080^**^ | 0.076^**^ | 0.080^**^ | 0.078^**^ | 0.080^**^ | 0.105^**^ | 0.086^**^ |
| Diff_GDP | 0.051 | 0.052 | - | 0.053 | 0.046 | 0.052 | 0.048 | 0.201^***^ | 0.046 |
| Diff_ GDP Per | 0.047 | 0.031 | 0.039 | - | 0.038 | 0.041 | 0.036 | 0.062^*^ | 0.038 |
| Diff_Population | -0.028 | -0.020 | -0.007 | -0.021 | - | -0.019 | -0.017 | 0.005 | -0.005 |
| Diff_Religion | -0.007 | -0.032 | -0.035 | -0.038 | -0.035 | - | -0.032 | -0.045 | -0.020 |
| Diff_Language | 0.051 | 0.052^*^ | 0.049 | 0.050^*^ | 0.051^*^ | 0.050^*^ | - | 0.045 | 0.056^*^ |
| Diff_Technology | 0.256^***^ | 0.294^***^ | 0.311^***^ | 0.289^***^ | 0.282^***^ | 0.286^***^ | 0.282^***^ | - | 0.302^***^ |
| Diff_IRCH organization | 0.095^**^ | 0.113^**^ | 0.107^**^ | 0.108^**^ | 0.106^**^ | 0.103^**^ | 0.110^**^ | 0.139^***^ | - |
| R^2^ | 0.143 | 0.196 | 0.200 | 0.200 | 0.202 | 0.201 | 0.199 | 0.152 | 0.191 |
| Adj-R^2^ | 0.134 | 0.188 | 0.192 | 0.192 | 0.193 | 0.193 | 0.191 | 0.144 | 0.183 |

^*^ p < 0.1, ^**^ p < 0.05 and ^***^ p < 0.01.

**Table A.3.** Results of the variable exclusion test in 2021.

| Variables | Model 1 | Model 2 | Model 3 | Model 4 | Model 5 | Model 6 | Model 7 | Model 8 | Model 9 |
| --- | --- | --- | --- | --- | --- | --- | --- | --- | --- |
| Diff_Geographic distance | - | -0.377^***^ | -0.354^***^ | -0.347^***^ | -0.333^***^ | -0.349^***^ | -0.353^***^ | -0.317^***^ | -0.335^***^ |
| Diff_Land borders | 0.197^***^ | - | 0.065^**^ | 0.072^**^ | 0.063^*^ | 0.063^*^ | 0.066^**^ | 0.086^**^ | 0.077^**^ |
| Diff_GDP | 0.093^**^ | 0.085^*^ | - | 0.097^**^ | 0.137^***^ | 0.082^*^ | 0.082^*^ | 0.222^***^ | 0.082^*^ |
| Diff_ GDP Per | -0.074^**^ | -0.093^**^ | -0.096^***^ | - | -0.076^**^ | -0.089^**^ | -0.088^**^ | -0.048 | -0.091^**^ |
| Diff_Population | 0.076^*^ | 0.126^***^ | 0.153^***^ | 0.116^**^ | - | 0.127^***^ | 0.127^***^ | 0.115^**^ | 0.140^***^ |
| Diff_Religion | 0.011 | -0.024 | -0.022 | -0.026 | -0.024 | - | -0.014 | -0.034 | -0.014 |
| Diff_Language | 0.042 | 0.033 | 0.023 | 0.026 | 0.029 | 0.017 | - | 0.028 | 0.033 |
| Diff_Technology | 0.198^***^ | 0.263^***^ | 0.297^***^ | 0.235^***^ | 0.249^***^ | 0.257^***^ | 0.256^***^ | - | 0.274^***^ |
| Diff_IRCH organization | 0.096^**^ | 0.139^***^ | 0.133^***^ | 0.135^***^ | 0.144^***^ | 0.131^***^ | 0.134^***^ | 0.157^***^ | - |
| R^2^ | 0.163 | 0.255 | 0.254 | 0.251 | 0.246 | 0.257 | 0.257 | 0.217 | 0.241 |
| Adj-R^2^ | 0.156 | 0.249 | 0.248 | 0.244 | 0.239 | 0.251 | 0.251 | 0.210 | 0.235 |

^*^ p < 0.1, ^**^ p < 0.05 and ^***^ p < 0.01.

**Table A.4.** The country codes.

| ISO3 Code | Country/Region Name | ISO3 Code | Country/Region Name | ISO3 Code | Country/Region Name |
| --- | --- | --- | --- | --- | --- |
| ARG | Argentina | GRC | Greece | NLD | Netherlands |
| AUS | Australia | GTM | Guatemala | NZL | New Zealand |
| AUT | Austria | HKG | China, Hong Kong SAR | PAK | Pakistan |
| BEL | Belize | HUN | Hungary | POL | Poland |
| BRA | Brazil | IDN | Indonesia | SEN | Senegal |
| CAN | Canada | IND | India | SGP | Singapore |
| CHE | Switzerland | IRL | Ireland | SLV | El Salvador |
| CHN | China | ITA | Italy | SVK | Slovakia |
| COL | Colombia | JAM | Jamaica | SVN | Slovenia |
| CZE | Czechia | JPN | Japan | SWE | Sweden |
| DEU | Germany | KOR | Rep. of Korea | SWZ | Eswatini |
| DNK | Denmark | LBN | Lebanon | THA | Thailand |
| EGY | Egypt | LVA | Latvia | UKR | Ukraine |
| ESP | Spain | MAR | Morocco | USA | United States |
| EU | European Union | MYS | Malaysia | VNM | Vietnam |
| FRA | France | NAM | Namibia | ZAF | South Africa |
| GBR | United Kingdom | NGA | Nigeria |  |  |
